# Is There a Causal Relationship between Intussusception and Food Allergy?

**DOI:** 10.3390/children4100089

**Published:** 2017-10-19

**Authors:** Emrah Aydin, Omer F. Beşer, Esra Ozek, Soner Sazak, Ensar Duras

**Affiliations:** 1 Department of Pediatric Surgery, Bahcelievler State Hospital, Kocasinan Karadeniz Mah. No. 48 Bahcelievler, Istanbul 34186, Turkey; 2 Department of Pediatric Gastroenterology, Okmeydani Education & Training Hospital, Istanbul 34384, Turkey; omerbeser@saglik.gov.tr; 3 Department of Pediatric Allergy and Immunology, Okmeydani Education & Training Hospital, Istanbul 34384, Turkey; esra.ozek@saglik.gov.tr; 4 Department of Pediatrics, Okmeydani Education & Training Hospital, Istanbul 34384, Turkey; soner.sazak@saglik.gov.tr (S.S.); ensar.duras@saglik.gov.tr (E.D.)

**Keywords:** intussusception, food allergy, recurrent intussusception

## Abstract

Although intussusception and food allergy are common health problems in childhood, the relation between these two diseases remain obscure. The aim of this study is to investigate the relationship between food allergy and intussusception, and the factors associated with both. Patients diagnosed with intussusception by the Brighton Collaboration Intussusception Working Group criteria were prospectively investigated for food allergy per the European Society for Pediatric Gastroenterology, Hepatology and Nutrition (ESPGHAN) Guideline. They were analyzed per demographic features, clinical, physical and laboratory findings. There were eight (38.1%) patients diagnosed with food allergy, while 13 (61.9%) patients were non-allergic. The mean number of days of presenting symptoms was 1.13 days in the allergy group and 7.85 days in the non-allergy group. The mean number of intussusception attacks was 1.63 in the allergy group while 1 in the non-allergy group (*p* < 0.05, relative risk (RR) = 2.6). In the allergy group, one (13%) patient was followed up, six (75%) patients were reduced with pneumatic and one (13%) patient reduced manually. In the non-allergy group, four (31%) patients were followed up, six (46%) patients were reduced with pneumotic, one (7%) patient was reduced manually, and resection anastomosis was performed in two (15%) patients. Food allergy is an unrecognized associated factor for intussusception patients, which increases the risk for recurrence. Due to the small patient population, these results should be interpreted with caution.

## 1. Introduction

Intussusception, which is the second most common abdominal emergency in children, occurs when a bowel segment invaginate as a telescope one into another. The incidence reported is 0.3–2.5 cases per 1000 live births throughout the world. The peak age is before two years of age, and males are affected three times more than females [1]. About 10% of cases have pathologic lead points which are an infection, as upper respiratory tract infection and gastroenteritis; congenital problems, such as Meckel diverticula and polyps; or malignancy, such as non-Hodgkin’s lymphoma [2,3]. Patients generally present with colicky abdominal pain, bilious vomiting, jelly blood stool, and palpable abdominal mass. The diagnosis of intussusception is determined mostly with ultrasonography and per the clinical case definition for the diagnosis of acute intussusception proposed by the Brighton Collaboration Intussusception Working Group [4]. Although there are surgical and nonsurgical treatment modalities, less than 5% of the cases might be reduced spontaneously without any intervention [5].

Food allergy is the adverse immunological reaction to a specific allergen, which is reproducible on repetitive exposure to the same allergen. The prevalence of food allergy has risen considerably in recent years, now estimated to be 5% in adults and 8% in children, with some regional variability [6,7,8]. Skin prick tests and serum specific immunoglobulin-E (IgE) tests can help to determine a diagnosis in accordance with the clinical history in patients. However, both tests are not required at the same time in all patients, and either can be sufficient in each patient [9,10]. An oral challenge test is usually necessary for the diagnosis of non-IgE-mediated allergy. Usually, a specific IgE test is negative in patients with gastrointestinal symptoms; however, the diagnosis of cow’s milk protein allergy (CMPA) should not be excluded, even if a specific IgE test is negative in patients who have skin lesions [11,12].

As the child’s age increases, the possibility of an organic cause of intussusception also increases, especially in cases with recurrent intussusception [13]. Rotavirus vaccination, lymphoid hyperplasia of the colon, enteric cyst, and Henoch Schonlein purpura are some known reasons of intussusception [14,15,16]. However, the etiology of the intussusception could not be shown in all cases. There are few case reports regarding the relation between intussusception and lymphoid hyperplasia due to food allergy [13,14]. Herein, the aim of this study is to demonstrate the incidence and type of food allergy in cases with recurrent and non-recurrent intussusception, the clinical features of the patients, and the factors to determine these. To our knowledge, this is the first study that demonstrates the relationship between intussusception and food allergy.

## 2. Materials and Methods

After the Institutional Review Board approval (ethical approval number is #2013-7659) was obtained, data were assembled through an institutional database and augmented with the electronic medical record for the hospital. Patients diagnosed with intussusception between January and December 2015 were prospectively invited for the study, of which 17 patients declined to participate. Patients were divided into two groups: diagnosed as allergy and not diagnosed as an allergy. Two groups were analyzed comparatively per demographic features, clinical, physical, and laboratory findings. 

**Figure 1:**
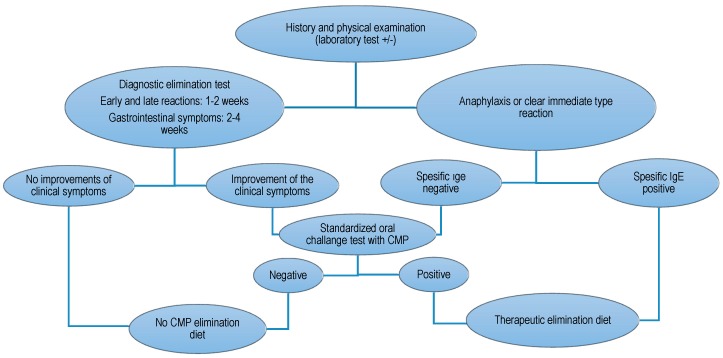
Algorithm for infants and children with symptoms suggestive of cow’s milk protein (CMP) allergy. IgE: immunoglobulin-E.

The diagnosis of intussusception was determined with abdominal graphs, ultrasonography, and per the clinical case definition for the diagnosis of acute intussusception proposed by the Brighton Collaboration Intussusception Working Group [4]. The diagnosis of food allergy was determined per the European Society for Pediatric Gastroenterology, Hepatology and Nutrition (ESPGHAN) Guideline: Diagnosis and Management of Food Allergy [10] (Figure 1). 

Eosinophilia was accepted as eosinophil values over 4% in complete blood count, and increased IgE was accepted as an increase in serum total IgE based on age-dependent values. Total IgE was tested nephelometrically using a BN2 nephelometer device (Siemens, Munich, Germany). Skin prick test (Allergopharma, Istanbul, Turkey) was performed in patients 3 years of age or older. The quick test applicator includes the allergens, which were histamine (positive control), negative control, dust mite (Der p and Der f), cat (*Felis domesticus*), dog (*Canis familiaris*), cockroach (*Blatella germanica*), grass pollen mix, mold mix (*Alternaria* and *Aspergillus*), cow milk, peanut, strawberry, tomato, chicken, banana, cocoa, wheat, and egg. It was performed at volar forearm. It took 20 min to check the response as induration. If the diameter of induration was over 3 mm, it was accepted as a positive response. A fluorescent enzyme immunoassay (FEIA) method was used for quantitative analysis of food-specific IgE using the UNICAP device (Phadia Austria GmbH Donau-City-Str. 1, Vienna, Austria). Food-specific IgE values higher than 0.35 kU/L were positive.

Weight, height, and length were measured using methods previously described by the World Health Organization (WHO) [17]. After measuring the weight, height, and length of all study population, four different *z*-scores (length/height-for-age, weight-for-age, weight-for-length/height, and body mass index-for-age) were calculated for children younger than 5 years old using WHO Anthro (Version 3.2.2, January 2011, Geneva, Switzerland, WHO) software. Body mass index-for-age (5–19 years old), height-for-age (5–19 years old), weight-for-age (5–10 years old) *z*-scores were calculated using WHO AnthroPlus software (Geneva, Switzerland, WHO) in children older than 5 years of age.

Statistical analysis was performed with IBM SPSS Statistics 20.0.0. The characteristics of the study sample were summarized by descriptive statistics, with dichotomous or ordinal data presented as percentages and continuous data as means with standard deviations. Kolmogorov–Smirnov test was used to demonstrate normal distribution. One-Way ANOVA was used for homogeneity of the variables. Student’s *t*-test and Pearson correlation were used for parametric data. Mann–Whitney U, Wilcoxon, and Kruskal–Wallis tests and Spearman correlation were used for non-parametric data. Statistical associations were considered significant if the *p*-value was <0.05.

## 3. Results

There were 38 patients diagnosed with intussusception in 2015. Twenty-one of them were included in the study. They were grouped into two as having a food allergy and not having a food allergy. Demographic and clinical features of the patients are presented in Table 1 and Table 2, respectively. Eight (38.1%) patients diagnosed with food allergy per ESPGHAN criteria while 13 (61.9%) patients were found to be non-allergic. Three (14.29%) of them had non-IgE type allergy, while five (23.81%) of them had IgE type allergy. The etiologic factors for the patients who were not allergic were upper respiratory tract infection in two patients (9.5%), acute gastroenteritis in one patient (4.8%), malignancy in two patients (9.5%) and idiopathic in eight patients (38.1%).

There was no statistical significance between groups by means of demographic and clinical features except the mean number of intussusception attacks. The mean number of days of presenting symptoms before admission to hospital was 1.13 in allergy group and 7.85 in the non-allergy group (95% confidence interval (CI) = 0.83–1.42, *p* = 0.098). One (12.5%) patient in the allergy group was diagnosed with ileoileal intussusception while four (30.77%) patients in the non-allergy group were diagnosed with ileoileal intussusception. 

In the allergy group, one (13%) patient was followed up, six (75%) patients were reduced with pneumotic, and one (13%) patient was reduced manually. In the non-allergy group, four (31%) patients were followed up, six (46%) patients were reduced with pneumotic, one (7%) patient was reduced manually, and resection anastomosis was performed in two (15%) patients. There was a statistical difference when patients compared per their need for an intervention (odds ratio (OR) = 0.321, 95% CI = 0.029–3.556, relative risk (RR) = 1.422 (0.769–0.2632)). 

Laboratory results of the groups are summarized in Table 3. The mean eosinophil count in the allergy group was 0.34/mm^3^, and 0.16/mm^3^ in the non-allergy group. In the allergy group, the mean of total IgE was 416.42 iU/mL (median = 123.61 iU/mL ± 846.06) and ECP was 58.31 µg/L (median = 44.55 µg/L ± 44.15). In the non-allergy group, the mean of total IgE was 102.74 iU/mL (median = 31.11 iU/mL ± 173.52) and eosinophilic cationic protein (ECP) was 28.47 µg/L (median = 19.20 µg/L ± 18.55). Specific IgE and/or skin prick tests were positive in five (62.5%) patients in allergy group, while they were negative for the rest of the study group. The allergens were cow milk in one patient, egg in two patients, wheat in one patient, and peanut and tomato in one patient. The number of days prior to admission to hospital was positively correlated with specific IgE values and negatively correlated with age of start for supplementary feeding.

**Figure 2:**
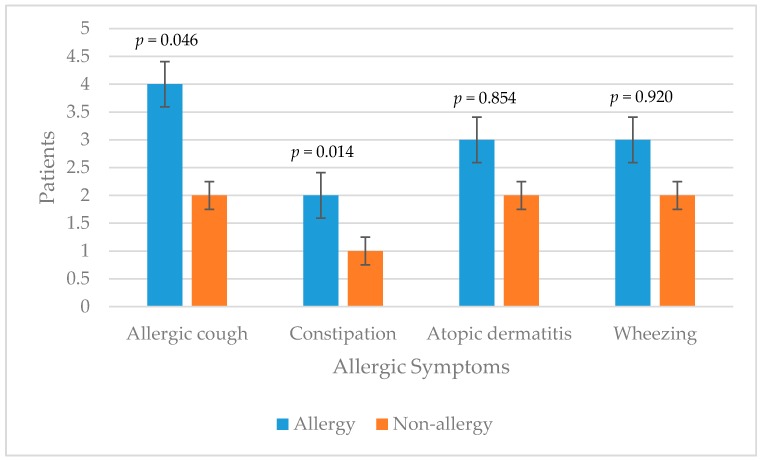
Distribution of the allergic symptoms between the groups.

In the allergy group, two patients were fed with breastfeeding for the first six months of life, five patients were fed between 6 and 12 months and one patient was fed more than 12 months. In this group, all patients were fed for the first six months only with breastfeeding. In the non-allergy group, two patients were fed with breastfeeding for the first six months of life, four patients were fed between 6 and 12 months and seven patients were fed more than 12 months. Ten patients were fed for the first six months only with breastfeeding, while three patients were fed between 6 and 12 months (*p* = 0.215, *p* = 0.163, respectively). In both groups patients had allergic symptoms prior to intussusception (Figure 2).

In the allergy group, the mean *z*-score weight for height was 0.19 (median = 0.05), weight for age was −0.47 (median = −0.67), height for age was −1 (median = −1.14) and BMI for age were −0.11 (median = 0.15). In the non-allergy group, the mean *z*-score weight for height was 0.27 (median = 0.23), weight for age was −0.21 (median = −0.02), height for age was −0.92 (median = −0.25) and BMI for age were 0.47 (median = 0.28) (*p* = 0.816, *p* = 0.578, *p* = 0.900, *p* = 0.103). 

## 4. Discussion

The main finding of the current study is the incidence of food allergy in patients diagnosed with recurrent intussusception is higher than the incidence in non-recurrent ones. The classical triad of abdominal pain, vomiting, and rectal bleeding as indicated in the textbooks results from invagination of the intestine one into another. There are few known causes of intussusception such as infection, malignancy, and congenital diseases, hence the etiology of many cases remains unresolved. On the other hand, food allergy, resulted by unfavorable and reproducible immunological reaction to a food, is a worldwide growing problem in childhood [6,7,8]. 

The findings of the study other than food allergy were correlated with the literature in means of etiologic factors and treatment strategies of intussusception [1,2,3,4]. The patient groups were alike with each other, except the mean number of intussusception attacks, which proves that the high incidence of food allergy was less likely to be an incidental finding. Food allergy was found in 38% of patients. The high incidence of food allergy might be the result of small patient population. However, it is important to demonstrate that food allergy is an etiologic factor, especially in recurrent intussusception, which was previously reported only as a few case reports [13,14]. 

While all patients in the non-allergy group had one intussusception attack, the mean intussusception attack in allergy group was 1.63, which was statistically significant. The time frame between attacks was 7–10 days (median = 7). A lymph node infiltrated by eosinophils due to allergens was speculated to be the underlying pathophysiologic mechanism that acted as a lead point in the allergy group and caused recurrent intussusception [13]. Even when the intussusception was treated, because the underlying reason was still there, it recurred. Patients with recurrent intussusception were free of symptoms after elimination of allergens from the diet. The provocation tests were positive in all patients, who had symptoms and signs of intussusception and were diagnosed with intussusception in a week after re-introduction of the allergens.

Even when patients diagnosed as lymphoma were excluded, patients in the non-allergy group were diagnosed with intussusception three times later after the first symptom than the patients in the allergy group. The hypersensitivity of these patients could be a reason for the acute onset of intussusception in this group. While not statistically significant, more patients had ileocecal intussusception, which may due to a high concentration of immune cells located at terminal ileum when compared to other parts of the intestine. It also explains the high percentage of need for intervention in allergy group when compared with the non-allergy group. The hyperreactivity in the immune system might have decreased the possibility of spontaneous reduction.

The mean eosinophil count and the mean of ECP were significantly higher in the allergy group. This correlates with the results of studies demonstrating the relationship between food allergy and these markers [18,19]. Allergens documented to cause intussusception were correlated with the ones in the literature, especially those found in IgE type food allergy [6,10,11,20]. The percentage of symptoms of atopy was also found higher in the allergy group, as correlated with literature [21,22]. A chronic cough and constipation were more common in statistically significant values, which proves the hypersensitivity in this group. 

Besides the known causes of intussusception, this study demonstrated that food allergy is a risk factor, especially in recurrent intussusception. Per the patient population was small and the study conducted in one country, multicenter studies with higher number of population will be needed to generalize these results. 

## Figures and Tables

**Table 1:** Demographic features of patients (*n* = 21).

	Allergy	Non-Allergy	*p*-Value
Age	4.36 ± 3.08	4.44 ± 3.10	0.955
Gender			0.864
Male	4 (50%)	7 (53.85%)	
Female	4 (50%)	6 (46.15%)	
Consanguineous marriage	4 (50%)	3 (23.08%)	0.204
Total breastfeeding			
<6 months	2 (25%)	2 (15.38%)	0.586
Supplementary feeding			
<6 months	5 (62.50%)	5 (38.46%)	0.284
Mean *z*-score			
Weight for height	0.19 ± 0.50	0.28 ± 0.61	0.816
Weight for age	−0.48 ± 1.14	−0.22 ± 0.95	0.578
Height for age	−1.01 ± 1.58	−0.93 ± 1.29	0.900
BMI for age	−0.11 ± 0.91	0.47 ± 0.66	0.103

Values expressed as means ± standard deviations (SDs) or count (percentage of group). BMI: body mass index.

**Table 2:** Clinical features of the patients.

	Allergy	Non-Allergy	*p*-Value
Physical examination			
Normal	2 (25%)	4 (30.77%)	0.776
X-ray			
Normal	2 (25%)	4 (30.77%)	0.776
Ultrasonography			
Intussusception	8 (100%)	8 (100%)	0.324
Level			
Ileoileal	2 (25%)	5 (38.46%)	0.525
Intervention	7 (87.50%)	9 (69.23%)	0.034
Number of intussusception attacks	1.63 ± 1.19	1 ± 0.0	0.039

Values expressed as means ± SDs or count (percentage of group).

**Table 3:** Laboratory results of the patients.

	Allergy	Non-Allergy	*p*-Value
Skin prick test	3 (37.50%)	1 (7.69%)	0.004
Eosinophils	0.34 ± 0.29	0.16 ± 0.19	0.014
Total IgE	416.42 ± 846.06	102.74 ± 173.52	0.205
ECP	58.31 ± 44.15	28.47 ± 18.55	0.043

Values expressed as means ± SDs or count (percentage of group). IgE: immunoglobulin-E; ECP: eosinophilic cationic protein.
